# Supplementary material for: Thioredoxin System Protein Expression in Carcinomas of the Pancreas, Distal Bile Duct, and Ampulla in the United Kingdom
Source: Diseases. 2024 Sep 24;12(10):227. doi: 10.3390/diseases12100227 (PMC11507029; doi:10.3390/diseases12100227)
Supplement: Supplementary file 1 [file diseases-12-00227-s001.zip › diseases-3172371-supplementary.pdf]

Supplementary Figure Legends

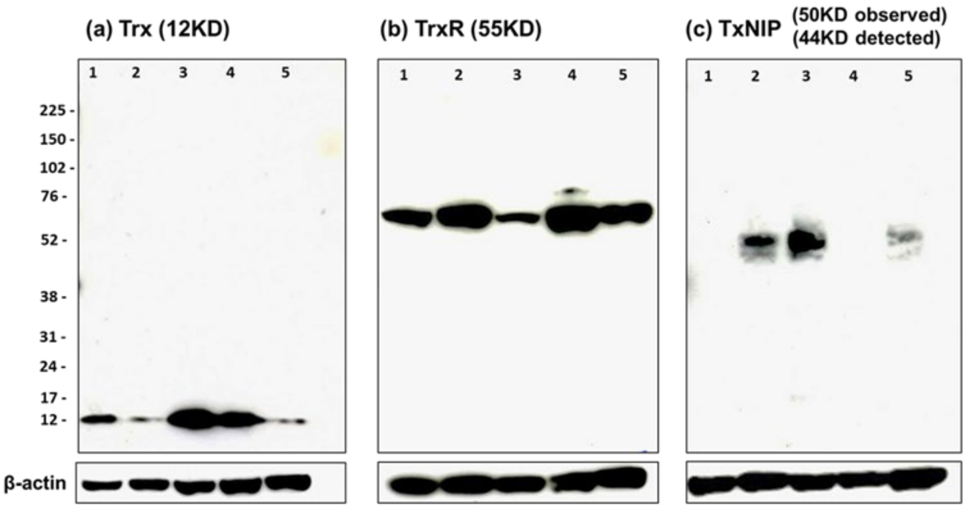

Figure S1. Specificity of Trx, TrxR, and TxNIP antibodies. Lysates from (1) PANC-1, (2) MIA Paca-2, (3) BxPc-3, (4) MDA-MB-231, and (5) MCF-7 cancer cell lines were subjected to ECL-western blot to assess the specificity of Trx (a), TrxR (b), and TxNIP (c) antibodies. The nitrocellulose membrane was incubated with a primary antibody at 4°C overnight and then with a secondary antibody at room temperature for 1 hour.

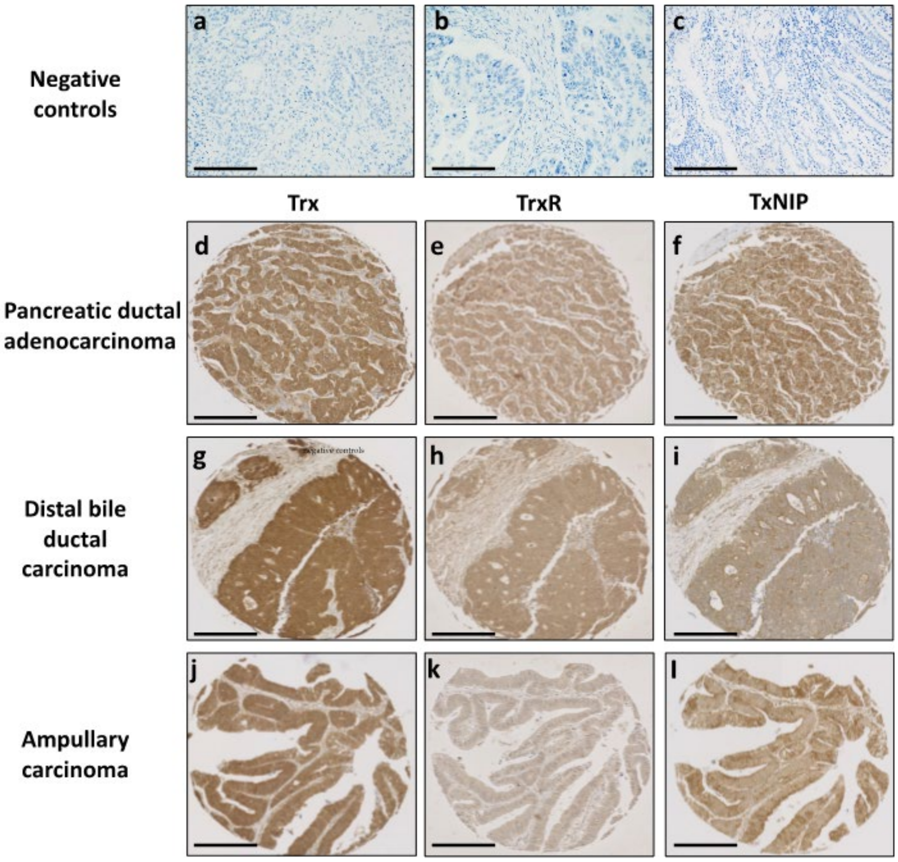

Figure S2. Representative photomicrographs of protein expression. Panels a, b, and c are negative controls of periampullary cancer tissue sections representing pancreatic ductal adenocarcinoma, distal bile ductal carcinoma, and ampullary carcinoma, respectively. Panels d, e, and f represent expressions of Trx, TrxR, and TxNIP in PDACs, respectively. Panels g, h, and i represent expressions of Trx, TrxR, and TxNIP in distal bile ductal carcinomas, respectively. Panels j, k, and l represent expressions of Trx, TrxR, and TxNIP in ampullary carcinomas, respectively. Photomicrographs are at 10× magnification; scale bar: 200 µm.

Table S1. Associations between Trx and TxNIP protein expression and various clinicopathological variables in the pancreatic ductal adenocarcinoma cohort. The number of observations for the cohort is shown for each clinicopathological variable; the table does not include the number of observations where clinicopathological data were not available. The frequency of observed clinicopathological variables is noted next to the variable subgroup. The P-values were calculated using Pearson chi square test of association (χ^2) or Fisher's exact test in a 2 × 2 table if a cell count was less than 5.

Variable	Trx (cytoplasmic)			Trx (nuclear)			TxNIP		
	Low	High	*p*-value	Low	High	*p*-value	Low	High	*p*-value
**Age**									
≤60 years	3 (3.9)	24 (31.6)	0.521	15 (20.0)	11 (14.7)	0.701	22 (27.2)	5 (6.2)	0.669
>60 years	9 (11.8)	40 (52.6)		26 (34.7)	23 (30.7)		46 (56.8)	8 (9.9)	
**Sex**									
Male	11 (14.3)	38 (49.4)	0.117	24 (31.6)	24 (31.6)	0.227	44 (53.0)	7 (8.4)	0.335
Female	2 (2.6)	26 (33.8)		18 (23.7)	10 (13.2)		25 (30.1)	7 (8.4)	
**Tumor size**									
≤2 cm	1 (1.3)	5 (6.6)	1.000	2 (2.7)	4 (5.3)	0.401	6 (7.4)	0 (0.0)	0.582
>2 cm	12 (15.8)	58 (76.3)		39 (52.0)	30 (40.0)		62 (76.5)	13 (16.0)	
**Tumor stage**									
1	1 (1.3)	0 (0)	0.063	1 (1.3)	0 (0.0)	0.057	1 (1.2)	0 (0.0)	0.398
2	1 (1.3)	16 (20.8)		12 (15.8)	4 (5.3)		17 (20.7)	1 (1.2)	
3	11 (14.3)	45 (58.4)		26 (34.2)	30 (39.5)		48 (58.5)	12 (14.6)	
4	0 (0.0)	3 (3.9)		3 (3.9)	0 (0.0)		3 (3.7)	0 (0.0)	
**Node status**									
Negative	4 (5.3)	21 (28.0)	1.000	13 (17.6)	12 (16.2)	0.674	25 (31.3)	3 (3.8)	0.526
Positive	9 (12.0)	41 (54.7)		28 (37.8)	21 (28.4)		42 (52.5)	10 (12.5)	
**Vascular invasion**									
Absent	4 (5.2)	22 (28.6)	1.000	17 (22.4)	8 (10.5)	0.118	24 (29.3)	5 (6.1)	0.799
Present	9 (11.7)	42 (54.5)		25 (32.9)	26 (34.2)		45 (54.9)	8 (9.8)	
**Perineural invasion**									
Absent	4 (5.2)	9 (11.7)	0.216	8 (10.5)	5 (6.6)	0.617	11 (13.4)	4 (4.9)	0.243
Present	9 (11.7)	55 (71.4)		34 (44.7)	29 (38.2)		58 (70.7)	9 (11.0)	

Table S2. Associations between TrxR protein expression and various clinicopathological variables in the pancreatic ductal adenocarcinoma cohort. The number of observations for the cohort is shown for each clinicopathological variable; the table does not include the number of observations where clinicopathological data were not available. The frequency of observed clinicopathological variables is noted next to the variable subgroup. The P-values were calculated using Pearson chi square test of association (χ^2) or Fisher's exact test in a 2 × 2 table if a cell count was less than 5. Significant P-values are indicated by *.

Variable	TrxR (cytoplasmic)			TrxR (nuclear)		
	**Low**	**High**	***p*-value**	**Low**	**High**	***p*-value**
**Age**						
≤60 years	9 (11.7)	18 (23.4)	0.565	9 (11.7)	18 (23.4)	0.457
>60 years	20 (26.0)	30 (39.0)		21 (27.3)	29 (37.7)	
**Sex**						
Male	19 (24.1)	31 (39.2)	0.995	19 (24.1)	31 (39.2)	0.767
Female	11 (13.9)	18 (22.8)		12 (15.2)	17 (21.5)	
**Tumor size**						
≤2 cm	2 (2.6)	4 (5.2)	1.000	3 (3.9)	3 (3.9)	0.680*
>2 cm	28 (36.4)	43 (55.8)		28 (36.4)	43 (55.8)	
**Tumor stage**						
1	1 (1.3)	0 (0.0)	0.304	1 (1.3)	0 (0.0)	0.228
2	6 (7.7)	11 (14.1)		6 (7.7)	11 (14.1)	
3	23 (25.9)	34 (43.6)		24 (30.8)	33 (42.3)	
4	0 (0.0)	3 (3.8)		0 (0.0)	3 (3.8)	
**Node status**						
Negative	15 (19.7)	12 (15.8)	0.033*	13 (17.1)	14 (18.4)	0.333
Positive	15 (19.7)	34 (44.7)		18 (23.7)	31 (40.8)	
**Vascular invasion**						
Absent	7 (9.0)	20 (25.6)	0.098	11 (14.1)	16 (20.5)	0.896
Present	23 (29.5)	28 (35.9)		20 (26.5)	31 (39.7)	
**Perineural invasion**						
Absent	5 (6.4)	9 (11.5)	0.816	5 (6.4)	9 (11.5)	0.734
Present	25 (32.1)	39 (50.0)		26 (33.3)	38 (48.7)	
